# Translation, Cross-Cultural Adaptation, and Psychometric Validation of the Chinese/Mandarin Cardiac Rehabilitation Barriers Scale (CRBS-C/M)

**DOI:** 10.1155/2021/5511426

**Published:** 2021-06-17

**Authors:** Xia Liu, Adeleke Fowokan, Sherry L. Grace, Biao Ding, Shu Meng, Xiu Chen, Yinghua Xia, Yaqing Zhang

**Affiliations:** ^1^Shanghai Jiao Tong University School of Nursing, Shanghai, China; ^2^KITE & Peter Munk Cardiac Centre, University Health Network, University of Toronto, Toronto, Ontario, Canada; ^3^Faculty of Health, York University, Toronto, Ontario, Canada; ^4^Shanghai Sixth People's Hospital, Shanghai, China; ^5^Xinhua Hospital Affiliated to Shanghai Jiaotong University School of Medicine, Shanghai, China

## Abstract

**Objective:**

Cardiovascular diseases are among the leading causes of morbidity in China and around the world. Cardiac rehabilitation (CR) effectively mitigates this burden; however, utilization is low. CR barriers in China have not been well characterized; this study sought to translate, cross-culturally adapt, and psychometrically validate the CR Barriers Scale in Chinese/Mandarin (CRBS-C/M).

**Methods:**

Independent translations of the 21-item CRBS were conducted by two bilingual health professionals, followed by back-translation. A Delphi process was undertaken with five experts to consider the semantics and cross-cultural relevance of the items. Following finalization, 380 cardiac patients from 11 hospitals in Shanghai were administered a validation survey including the translated CRBS. Following exploratory and confirmatory factor analysis, internal consistency was assessed. Validity was tested through assessing the association of the CRBS-C/M with the CR Information Awareness Questionnaire.

**Results:**

Items were refined and finalized. Factor analysis of CRBS-C/M (Kaiser Meyer Olkin = 0.867, Bartlett's test *p* < 0.001) revealed five factors: perceived CR need, external logistical factors, time conflicts, program and health system-level factors, and comorbidities/lack of vitality; Cronbach's alpha (*α*) of the subscales ranged from 0.67 to 0.82. The mean total CRBS score was significantly lower in patients who participated in CR compared with those who did not, demonstrating criterion validity (2.35 ± 0.71 vs. 3.08 ± 0.55; *p* < 0.001). Construct validity was supported by the significant associations between total CRBS scores and CR awareness, sex, living situation, city size, income, diagnosis/procedure, disease severity, and several risk factors (all *p* < 0.05).

**Conclusions:**

CRBS-C/M is reliable and valid, so barriers can be identified and mitigated in Mandarin-speaking patients.

## 1. Background

Cardiovascular diseases (CVDs) remain a leading contributor to morbidity and mortality worldwide and exert a significant toll on health systems [1, 2]. While CVDs have been in decline in high-income countries, the opposite is true in low- to middle-income countries (LMICs) [3], with about 75% of CVD deaths occurring in LMICs [4]. Specifically in the LMIC of China, for example, the prevalence of CVDs rose from about 40.6 million to about 93.8 million from 1990 to 2016, resulting in a concomitant increase in CVD-related mortality from 2.51 million to 3.97 million within the same time period [5].

Cardiac rehabilitation (CR) is a comprehensive outpatient chronic disease management program delivering all guideline recommendations for secondary prevention [6]. The benefits of CR participation are well established, including reduced hospital readmissions and CVD mortality rates [7, 8]. Despite these benefits, CR utilization is low around the world [9, 10]. The reasons have been well characterized in high-resource settings, including patient-related factors (e.g., support, transportation access, and perceived need based on clinical status), provider-level factors (e.g., lack of patient referral), and system-level factors (e.g., lack of coverage and lack of programs) [11].

Of all countries globally, the second greatest unmet need for CR exists in China [12]. Therefore, we must address the barriers to use. With only 216 programs available across the entire country [13], clearly more programs are needed, and indeed, according to the Chinese Association of Cardiovascular Prevention and Rehabilitation, they are initiating new programs at an incredible rate. Again, clearly, we must understand barriers to use in this setting so they can be addressed through the design of the programs being built. However, in a review of studies on CR barriers in LMICs [14], only 1 study in China was identified, which only focused on healthcare providers' perceptions of barriers [15]. More recent work has examined CR guidance to inpatients, and disparities in CR utilization by age and sex were reported [16], as is reported broadly [10]. There has been minimal research on CR barriers published in the Chinese language as well [17, 18].

Validated scales such as the Cardiac Rehabilitation Barriers Scale (CRBS) are key to identifying these barriers at multiple levels [19] so they can be mitigated. CRBS has now been translated to 14 languages, including languages spoken in some lower-income countries such as Persian, Malay, Marathi, Hindi, Spanish, and Thai (see https://sgrace.info.yorku.ca/cr-barriers-scale/crbs-instructions-and-languages-translations/). Studies in nondeveloped countries have revealed that the key barriers can differ from those identified in high-income countries [20–22], underscoring the need to assess barriers specific to context. A simplified Chinese version of CRBS was recently published [23], although there were some limitations to the translation and validation process. For instance, only 1 person was involved in the translation and back-translation, 2 items were deleted without a sound reason, it was only validated in 126 patients from a single center, there was no confirmatory factor analysis of the subscale structure, and no test of validity.

Accordingly, the aim of this study was to (1) rigorously translate and cross-culturally adapt CRBS to Mandarin (simplified Chinese) using best practices and then (2) psychometrically validate the translation. This involved assessing factor structure (including confirmatory factor analysis), reliability (internal), and validity (criterion and construct). The final aim (3) was to identify the main barriers in the population.

## 2. Methods

### 2.1. Design and Procedure

This was a multimethod study.

#### 2.1.1. CRBS Translation and Cultural Adaptation

The multistep process of translation and cultural adaptation was done in accordance with best practices [24–27]. The initial translation of the scale from English to the target language (Mandarin; simple Chinese) was performed by two health professionals (a nurse researcher and a cardiologist) independently, both fluent in English, whose mother tongue was Mandarin. After the two translations had been performed, the first author combined them for consideration by an expert in rehabilitation. This first version of the instruments was then back-translated into English by two medical professionals who were not provided the original English version, resulting in the second version.

Next, a review committee which was comprised of five experts in the field of CR (two cardiomedical experts, one rehabilitation expert, and two nursing professors) was engaged in a Delphi process. There were two rounds of consultations to verify semantic and cross-cultural relevance of the items. Finally, the back-translated and revised version of the scale was then compared with the original version to consider conceptual discrepancies, after which the Chinese/Mandarin version of CRBS was finalized.

The 5 experts were asked to rate the content validity in both rounds, to establish that CRBS-C/M had an appropriate sample of items for CR barriers; the content validity index (CVI) for the items and scale were computed.

#### 2.1.2. Psychometric Validation

Participants for this study were recruited from outpatient cardiology clinics and wards in 11 hospitals in Shanghai, China. Four research nurses (in groups of 2) went to the hospitals between September and November 2017 on weekdays to collect data. Participants were first briefly informed of the purpose and significance of the study, after which informed consent was obtained. The participants then completed the paper-based questionnaire on site.

### 2.2. Setting and Participants

Two of the 11 hospitals have CR programs to which patients could be referred locally. The CR involved structured, supervised exercise sessions and patient education. The latter was delivered via hard copy education materials (including text and pictures) 1-1 to the patient.

Patients diagnosed with new myocardial infarction (including silent infarcts identified through an electrocardiogram) or acute coronary syndrome, chronic stable angina, heart failure, or having had coronary artery bypass surgery, percutaneous coronary intervention, and/or valvular surgery were eligible for the study. The inclusion criterion was age 18-75 years. Those with severe comorbidities were excluded. If the participants had low literacy and hence could not read or write in Mandarin, the research nurse would read the questionnaire item by item and denote their responses.

### 2.3. Measures

The survey was in Mandarin. The survey commenced with items regarding sociodemographic (e.g., age, sex, marital status, living arrangements, education, work status, and healthcare coverage) as well as clinical (e.g., diagnosis/procedures, duration of disease, disease severity, risk factors, comorbidities, and heart health behaviors) characteristics. To assess criterion validity, CR participation (any; yes/no) was collected via self-report as well.

#### 2.3.1. Cardiac Rehabilitation Barriers Scale

The CRBS scale evaluated patients' perception of the degree to which patient, provider, and health system-level barriers affect their CR enrolment and participation (i.e., all items applicable to enrollees and nonenrollees alike). The English version consists of 21 items (barriers) related to 4 subscales: perceived need/healthcare system factors, logistical barriers, work/time conflicts, and comorbidities/functional status [19] (although some translations consist of 5 subscales) [22]. Items are rated on a five-point Likert scale (1—strongly disagree to 5—strongly agree). Higher scores indicate greater barriers to CR. Criterion validity has been established in the English version and in many of the translated versions, demonstrated by significant differences in CRBS scores by CR use [19–21]. Reliability is also established [19].

Where participants completed more than 80% of the items, a mean total score was computed. Subscale scores were also computed based on the results of the factor analysis.

#### 2.3.2. Items Administered to Assess Construct Validity

In addition to the sociodemographic and clinical characteristics assessed above, 2 psychometrically validated scales were administered to assess construct validity. The Hospital Anxiety and Depression Scale (HADS) is a 14-item questionnaire used to screen for psychosocial distress in general hospital outpatients [28]. HADS consists of a 7-item anxiety subscale and a 7-item depression subscale. Respondents rate each item from 0 (not at all) to 3 (most of the time). The sum of the 7 items in each subscale is computed, with higher scores denoting greater symptoms and scores > 7 denoting “elevated” symptoms. The reliability and validity of the Chinese version of the HADS scale have been previously established [29].

The Cardiac Rehabilitation Information Awareness Questionnaire (CRIAQ) was developed by Jing [30] to assess CR and secondary prevention-related knowledge in patients with heart disease. Items were based on the American College of Cardiology, American Heart Association, and the American Cardiovascular and Pulmonary Rehabilitation Association guidelines and the 2013 Chinese consensus statement on CR [31–33]. The 29-item scale assesses basic awareness of CR and its components as well as risk factor control, using multiple choice response options. Correct responses are allotted 1 point (questions with multiple correct response options are assigned 1 point for each correct response), while incorrect answers and “do not know” responses are not given any points. The maximum total sum score is 93. Higher scores indicate better awareness of CR and secondary prevention.

### 2.4. Data Analyses

The Statistical Package for Social Sciences v. 23 (SPSS Inc., Chicago, IL, USA) was used for all data analysis, except that the lavaan version (0.6-5) in R 3.6.2 was used for confirmatory factor analysis (CFA). The level of significance for all tests was set at 0.05. A descriptive examination of participant characteristics, as well as CRBS and CRIAQ items, was performed.

For the psychometric validation, first exploratory factor analysis (EFA) was performed. Factor extraction was conducted using the principal component method with varimax rotation. The number of factors to extract was determined by examining the scree plots and considering factors with eigenvalues greater than 1.0. Item factor loadings > 0.3 were considered in finalizing the items for each factor and interpreting the factors [34].

CFA was then done to verify the factor structure obtained from EFA. To determine adequacy of fit, indices considered were the model chi-square/df index, the comparative fit index (CFI), the Tucker-Lewis index (TLI), the Akaike information criterion (AIC), the Bayesian information criterion (BIC), the standardized root mean square residual (SRMSR), and the root mean square error of approximation (RMSEA); *χ*^2^/df values less than 4, CFI and TLI values greater than 0.9, and RMSEA values ≤ 0.06 were considered indicative of a good fit [35].

To determine the internal consistency, Cronbach's *α*values of the scale and subscales were calculated. In this analysis, *α* values greater than 0.70 were considered acceptable [36], reflecting the correlation of the items among themselves and with the total score. Cronbach's *α* values if each item were deleted were checked to determine if internal reliability would improve if any items were deleted from the scale.

To assess criterion validity, differences in CRBS item, subscale, and total scores by CR participation were tested using Student's independent samples *t*-tests. To assess construct validity, Pearson's correlation, independent samples *t*-tests, and analysis of variance were used to explore associations between sociodemographic and clinical characteristics of study participants as well total CRIAQ scores with mean total CRBS score, as applicable.

## 3. Results

### 3.1. Translation and Cultural Adaptation

Following translations and harmonization of CRBS to Mandarin, through the Delphi process, the expert health professionals deemed all 21 questions in the original CRBS version applicable to the Chinese context, but suggested more detail be added to some of the questions. Hence, slight changes were made to some of the questions. For example, to CRBS item 1 “…of distance,” they added “(e.g., not located in your area, too far to travel).” CRBS 18 was modified from “I can manage on my own” to “I can manage my heart problem on my own.” The committee also agreed to revise some of the items to aid clarity. Modifications were made to CRBS items 2-6, 10-11, 17, and 21 without changing their semantic value. They also considered adding some additional items (i.e., duration of program, duration of sessions, did not perceive benefit from sessions, and went to a few sessions and feel they can do the exercise independently); however, they would only be relevant to CR enrollees and therefore they were not incorporated. The I-CVIs ranged from 0.80 to 1.00, and the S-CVI was 0.92, which establishes that the Chinese version of CRBS has acceptable content validity. The final C/M survey is shown in the online supplement.

### 3.2. Participant Characteristics

The sample was comprised of 380 participants, of which 19 (5.0%) participated in CR (Table 1). As shown, CR participants were more likely to live alone, had lower family income, were more likely to have a diagnosis of stable angina or others, and to have bypass surgery (but less likely to have had percutaneous coronary intervention) than patients who did not participate in CR. CR information awareness among those who did not participate in CR is shown in Table 2.

### 3.3. Psychometric Validation

The structure of the scale was first assessed using principal component analysis. The Kaiser Meyer Olkin value was 0.867, and Bartlett's test was significant (*p* < 0.001), highlighting the suitability of our data for factor analysis. Five components with eigenvalues greater than 1.0 were obtained. These factors, considered together, accounted for 59.2% of the total variance.  Table 3 displays the eigenvalues and the variance explained by each factor.

CRBS item factor loadings are also shown in Table 3. The first factor reflects perceived CR need. The second factor reflects external logistical factors that impede access such as transportation, distance, and cost. The third factor reflects time conflicts that impede access such as travel, work, and family responsibilities. The fourth factor reflects program and health system-level factors. Lastly, the fifth factor reflects comorbidities/functional status. The first three factors of the Chinese/Mandarin version of CRBS showed good internal consistency (Cronbach′s *α* ≥ 0.7; Table 3). However, internal consistency of factors 4 and 5 fell slightly short of the 0.7 threshold.

The model fit indices for the CFA were found to be acceptable with a chi-square/df of 2.66, a TLI of 0.872, a CFI of 0.896, an SRMSR of 0.054, and an RMSEA of 0.066 (90%confidence intervals = 0.059 to 0.074). The estimates showed significant factor loadings (*p* < 0.001 for all), with factor loadings which ranged from 0.29 to 0.90, documenting that the items were a good fit to the scale.

With regard to criterion validity, Table 4 displays the mean item, subscale, and total CRBS scores by participation in CR. Patients who participated in CR reported significantly lower mean total CRBS scores than those who did not; all subscale scores and individual scores for items 1-9 (trend for 10 and 11), 13, and 15-21 were significantly lower in CR participants.

With regard to construct validity, a significant negative association was observed between the CRIAQ and the CRBS scales (*p* < 0.001) (Table 1; significant correlations between almost all CRBS items and total CRIAQ scores are shown in Supplemental Table 1). In further support of construct validity, significant associations were observed between sex (women reported more barriers), living status (those living alone reporting more barriers), residence (those living outside a city or town reporting more barriers), diagnosis (those with unstable angina and “other” categories reporting more barriers), coronary artery bypass grafting (those having this reporting less barriers), body mass index (those more overweight reporting fewer barriers), tobacco use (never users reporting more barriers), family history of CVD (those with a family history reporting fewer barriers), regular exercise (those with an exercise history reporting fewer barriers), and sodium intake (those with lower intake reporting lower barriers) with mean CRBS total score (Table 1).

### 3.4. Main Barriers

The main barriers in the nonenrollees were distance, lack of awareness, weather, and transportation (Table 4). In CR participants, these were comorbidities, distance, weather, lack of physician encouragement, and time constraints.

## 4. Discussion

This study sought to rigorously translate, cross-culturally adapt, and psychometrically validate CRBS into Chinese/Mandarin. Through this process, all 21 items of the scale were retained, with slight adjustments made to some items to improve clarity. Factor analysis revealed five factors: CR need, external logistical factors, time conflicts, program and health system-level factors, and comorbidities/functional status. The subscales showed relatively good internal consistency (reliability). The significantly lower mean CRBS scores in patients who participated in CR establish the criterion validity of CRBS. Construct validity was demonstrated by significant associations between CRBS scores and many sociodemographic and clinical characteristics known to impact CR access, but surprisingly not anxious and depressive symptoms [37]. Lastly, the consistent associations observed between CRBS and the CRIAQ scale further underscore the construct validity of CRBS. Taken together, the results from this study establish the validity and reliability of the Chinese/Mandarin version of CRBS in assessing barriers to CR.

There are some differences in this C/M version of CRBS and the previously published one (named “C”) [23]. Some concerns regarding the development of CRBS-C were outlined in Background. Importantly, information about the participants and hospital was not fulsomely reported, and therefore generalizability is unclear. Furthermore, it was not stated whether there was a CR program at the hospital where the patients were surveyed and whether there was CR attendance by the patients, which would greatly impact ratings and mean scores.

Second, in CRBS-C [23] but not in C/M, items 19 and 20 were deleted. While through our scientific process deletion of items was also considered, whether the 2 items should have been deleted from the C version based on issues raised in the focus groups is questionable. Item 19 (“I think I was referred but the rehab program didn't contact me”) was deleted “considering there is no CR center and corresponding referral system in China.” It may be that there was no CR at the hospital where the study was undertaken (as raised above), but this is not true across China [38]. The score for item 20 (“it took too long to get referred and into the program”) was quite high ((3.28 ± 1.52)/5), yet it was deleted because while “the vast majority of patients had this obstacle, there is a lack of CR recognition.”

Finally, there were similarly 5 factors identified in the remaining 19 items, namely, time/work conflicts (items 12, 11, 13, and 10), cost/travel (items 1, 3, and 2), CR need (items 7, 18, and 6), physical/function limitations (items 15, 14, 16, and 8), and lack of CR knowledge (items 5, 17, 4, 20, and 9). These are quite similar, except that the latter was “program and health system-level factors” in our CRBS-C/M version. Although overall the psychometric properties of CRBS-C were favourable, some items were loaded onto factors in a way that would not be expected. For example, item 8 “severe weather” loaded onto the “Physical/functional limitation” factor (but on the “external logistical” factor in this version), and item 4 “family responsibilities” loaded onto the factor “Lack of CR knowledge” (but on the “time conflicts” factor in this version). Arguably, the item loadings overall on each factor are a better fit in the C/M version.

There are now 14 translations of CRBS, and with this version, and of those translations for which factor structure has been tested, 3 of them similarly have 5 rather than 4 factors as per the original version [19], namely, the Brazilian-Portuguese [22] and Turkish versions [39]; the Korean version has 6 [20] (the Malay version also has 4) [21]. In the English version, the perceived need and healthcare items were bundled onto one factor; they were separate factors in the Chinese/Mandarin version, suggesting the structure is fairly consistent across languages and cultures. Given the differential responses in enrollees and nonenrollees and the likely different proportion of enrollees and nonenrollees in the cohorts across these validations, more research may be needed to test the factor structure in enrollees vs. nonenrollees. Indeed, in the current sample, there were very few enrollees; future research is needed to assess whether the scale is valid in that population, and to better understand their barriers to program adherence.

In this sample, 90% of participants had not even heard of CR before the study. Certainly, lack of awareness was a key barrier to CR utilization. Consistent with previous research [40], their awareness of secondary prevention strategies was also lower than needed for patients to achieve risk reduction (with 90% not even knowing what secondary prevention is). For instance, 1/5 did not know what CR was comprised of, and only 1/4‐1/3 were aware of some major benefits of CR, including improvements in quality of life. One-third to 1/2 were not aware of some of the major CVD risk factors, including physical inactivity, stress, tobacco use, and obesity, and 1/3 did not know whether CVD could be controlled; clearly, these patients then would not be in a position to self-manage and reduce their risk of recurrence. Half did not know about the importance of taking lipid-lowering drugs, two-thirds did not know that reducing sodium intake could reduce blood pressure, 80% perceived that they could stop antihypertensive medication once their blood pressure was under control, and almost half thought that they should stay in bed to control their blood pressure. The majority of patients did not know the healthy waist circumference target. Finally, Chinese patients were not familiar with the recommended modes of exercise or intensity. We recently translated and cross-culturally adapted an evidence-based CR patient education curriculum (https://www.healtheuniversity.ca/zh/cardiaccollege/Pages/default.aspx) [41, 42], so that hopefully these information gaps can be overcome.

The implications of this work are that now that we can validly and reliably assess CR barriers in Chinese samples, we can work to identify and mitigate them. One of the key barriers in nonenrollees was lack of awareness. Considering most patients were recruited at hospitals without CR programs, it is not surprising that their healthcare providers did not inform them about CR services; however, patients should have been educated about secondary prevention strategies at the least. As mentioned, with a burgeoning number of CR programs being built, this situation will change. Given that there is no reimbursement for CR services [43] and because of the sheer number of CVD patients [12], it will take much effort to ensure sufficient CR capacity in China.

Other key barriers, namely, distance, weather, and transportation, could be mitigated with the provision of home-based CR, potentially exploiting technology such as WeChat which is so popular in China. Unfortunately, only 17% of the programs in China currently offered home-based services [44]. Moreover, as outlined above, barriers in enrollees warrant further research attention, so programs can be adapted to address them.

Finally, as shown in previous literature [45], certain patients had greater barriers, such as women, those living alone (and hence having less support), or those living outside the city. CR programs could be modified to attempt to address barriers in these vulnerable groups. Moreover, as in other literature, it appears clinical diagnosis confers some barriers, such as for example patients who have had bypass surgery being clearly indicated based on benefit [46], and likely more strongly encouraged to go, reducing barriers [10]. In this sample, associations with health behaviors were also found (although the association with tobacco use was opposite surprisingly), such that patients who engage in healthier lifestyles reported fewer barriers; this is likely due to a “third variable” of conscientiousness or socioeconomic status.

### 4.1. Study Limitations

Caution is warranted in interpreting these results. First, only a small percentage (5%) of the sample participated in CR, and therefore results are primarily generalizable to those who do not access CR (which is the majority) [11]. Moreover, the findings may not be generalizable to patients outside of Shanghai in China. Second, cognitive debriefing was not undertaken with patients. Third, the internal reliability of the last 2 factors was weaker, which should be considered more closely in future research. Fourth, multiple comparisons may have increased the likelihood of a Type 1 error. Finally, due to the nature of the design, causal conclusions cannot be drawn.

## 5. Conclusion

The Chinese/Mandarin version of CRBS was developed, and its structure is comprised of five subscales, namely, perceived CR need, external logistical factors, time conflicts, program and health system-related factors, and comorbidities/functional status. It was found to have good psychometric properties, underscoring its reliability and validity in assessing barriers to CR utilization in Chinese individuals. This scale will be vital in identifying barriers so we can improve utilization in places with CR programs, by addressing the key barriers identified.

## Figures and Tables

**Table 1:** Sociodemographic and clinical characteristics of study participants by CR participation, and association with total CRBS score.

	Total *n* = 380	Participated in CR *n* = 19	Did not participate *n* = 361	*p* ^∗^	*p* ^†^
*Sociodemographic*					
Age	67.02 ± 11.09	62.3 ± 8.97	67.29 ± 10.87	0.079	0.240
Sex (% female)	128 (37%)	8 (42.1%)	120 (37.0%)	0.657	0.004
Marital status (% married)	331 (86%)	18 (94.7%)	309 (95.4%)	0.899	0.875
Living status (% alone)	59 (15.3%)	1 (5.3%)	58 (17.8%)	0.157	0.010
Caregivers				0.001	0.089
Family (e.g., spouse and child)	193 (50.2%)	9 (47.4%)	180 (55.4%)		
Self	153 (39.7%)	10 (52.6%)	143 (44.0%)		
Nurse	2 (0.5%)	0	2 (0.6%)		
Nationality (% Han)	346 (89.9%)	19 (100%)	323 (99.4%)	0.732	0.241
Education				0.367	0.152
Junior high school and below	159 (46.2%)	8 (42.1%)	151 (46.5%)		
Technical secondary school/senior high school	133 (38.7%)	6 (31.6%)	127 (39.1%)		
College degree	52 (15.1%)	5 (26.3%)	47 (14.5%)		
Work status (% working)	54 (15.7%)	6 (31.6%)	48 (14.8%)	0.051	0.467
Residence (% city or town)	327 (84.9%)	19 (100%)	305 (94.1%)	0.277	0.020
Family income				0.029	0.112
(% >40001RMB)^‡^	191 (49.6%)	10 (52.6%)	181 (55.7%)		
≤40000RMB	153 (50.4%)	9 (47.4%)	144 (44.3%)		
Healthcare coverage				0.306	0.263
Insurance or government	330 (85.7%)	19 (100%)	307 (94.8%)		
Out-of-pocket	17 (4.4%)	0	17 (5.2%)		
*Clinical*					
Duration of CHD				0.975	0.839
<1 year	127 (33%)	7 (36.8%)	120 (37.2%)		
1-5 years	104 (27%)	5 (26.3%)	96 (29.7%)		
>5 years	114 (29.6%)	7 (36.8%)	106 (32.8%)		
Diagnosis				0.041	<0.001
Silent infarction	159 (46.6%)	7 (36.8%)	152 (47.2%)		
Myocardial infarction	91 (26.7%)	5 (26.3%)	86 (26.7%)		
Other	40 (11.7%)	4 (21.1%)	36 (11.2%)		
Unstable angina	36 (10.6%)	0	36 (11.2%)		
Stable angina	15 (4.4%)	3 (15.8%)	12 (3.7%)		
PCI (% yes)	264 (78.3%)	8 (42.1%)	256 (80.5%)	<0.001	0.757
CABG (% yes)	14 (3.6%)	6 (31.6%)	8 (2.6%)	<0.001	<0.001
Medication adherence (% regularly taking)	304 (79%)	18 (94.7%)	285 (95%)	0.394	0.553
NYHA class				0.980	0.065
I	163 (42.3%)	9 (47.4%)	154 (47.5%)		
II	143 (37.1%)	8 (42.1%)	135 (41.7%)		
III	35 (9.1%)	2 (10.5%)	32 (9.9%)		
IV	3 (0.8%)	0	3 (0.9%)		
*Risk factors*					
BMI	24.33 ± 3.43	24.43 ± 2.82	24.35 ± 3.45	0.920	0.032
Tobacco use				0.261	0.002
Never used	176 (51.2%)	12 (63.2%)	164 (50.5%)		
Former user	101 (29.4%)	6 (31.6%)	95 (29.2%)		
Current user	67 (19.5%)	1 (5.3%)	66 (20.3%)		
Hypertension (% yes)	245 (71.4%)	15 (78.9%)	230 (71%)	0.455	0.071
Diabetes (% yes)	124 (36.2%)	9 (47.4%)	115 (35.5%)	0.295	0.639
Family history of CVD	146 (42.6)	10 (52.6%)	136 (42%)	0.361	0.042
*Comorbidities*					
Stroke	19 (4.9%)	1 (5.3%)	18 (5.6%)	0.957	0.764
Renal insufficiency	11 (2.9%)	0	11 (3.4%)	0.414	0.793
Transient ischemic attack	4 (1.0%)	0	4 (1.2%)	0.626	0.169
Peripheral vascular disease	6 (1.6%)	1 (5.3%)	5 (1.5%)	0.229	0.279
*Health behaviors*					
Harmful use of alcohol (% ≥2 drinks/day)	2 (0.5%)	0	2 (0.6%)	0.766	0.731
Regular exercise (% ≥3 times/wk for ≥30 min)	200 (52%)	18 (94.7%)	182 (56.0%)	0.001	<0.001
Monthly sodium intake^§^				0.050	0.001
<120 g	22 (5.7%)	0	22 (6.8%)		
120-179 g	229 (59.5%)	14 (73.7%)	214 (65.8%)		
>180 g	94 (24.4%)	5 (26.4%)	89 (27.4%)		
Daily fruit intake°				0.533	0.287
<50 g	42 (10.9%)	1 (12.5%)	40 (20.1%)		
50-200 g	151 (39.2%)	6 (87.5%)	142 (71.3%)		
>200 g	17 (4.4%)	0	17 (8.5%)		
*Psychosocial*					
HADS					
Depressive symptoms	9.85 ± 1.87	10.28 ± 1.90	9.82 ± 1.87	0.323	0.227
Anxiety	13.64 ± 1.78	13.39 ± 2.03	13.66 ± 1.76	0.525	0.532
CR information awareness	46.4 ± 12.5	63.6 ± 17.1	45.4 ± 11.5	<0.001	<0.001

^‡^$1 USD = 7RMB. ^§^Based on recommendations of 3-5 grams/day; ideally participants would have <150 grams/month. °~>400 g/day recommended (e.g., 5 servings of 80 grams). ^∗^*p* is used for comparison of variables by CR participation status, using chi-square or *t*-test, as appropriate. ^†^*p* is used for association of variable value in total sample with total mean CRBS score, tested using independent sample *t*-test, analysis of variance, or Pearson's correlation, as applicable. BMI: body mass index; CR: cardiac rehabilitation; CRBS: Cardiac Rehabilitation Barriers Scale; CHD: coronary heart disease; PCI: percutaneous coronary intervention; CABG: coronary artery bypass grafting; NYHA: New York Heart Association; CVD: cardiovascular disease; HADS: Hospital Anxiety and Depression Scale. Note: some data were missing, and therefore the percentage reported was based on the available denominator.

**Table 2:** Cardiac Rehabilitation Information Awareness Questionnaire responses in those not participating in CR, *n* = 361.

Item	Frequency (%)
(1) Have you ever heard of cardiac rehabilitation before this survey?	
(a) Yes	31 (9.5%)
(b) No	294 (90.5%)
(2) Which of the following should be included in cardiac rehabilitation? (check all that apply)^˄^	
(a) Illness assessment	11 (35.5%)
(b) Lipid management	17 (54.8%)
(c) Hypertension management	17 (54.8%)
(d) Tobacco cessation/alcohol restriction	16 (51.6%)
(e) Diabetes management	11 (35.5%)
(f) Nutrition consultation	10 (32.3%)
(g) Weight management	15 (48.4%)
(h) Emotional regulation	12 (38.7%)
(i) Physical activity consultation	9 (29.0%)
(j) Exercise training	12 (38.7%)
(k) Sleep management	14 (45.2%)
(l) Regular follow-up	14 (45.2%)
(m) Medication review	15 (48.4%)
(n) I do not know^∗^	7 (22.6%)
(3) Which of the following are benefits of participating in cardiac rehabilitation? (check all that apply)^˄^	
(a) Cure coronary heart disease^∗^	11 (35.5%)
(b) Improve cardiac function	19 (61.3%)
(c) Reduce acute ischemic coronary events	12 (38.7%)
(d) Reduce mortality and recurrence of cardiovascular disease	16 (51.6%)
(e) Save medical expenses	7 (22.6%)
(f) Improve quality of life	10 (32.3%)
(g) Help to return to family and society	8 (25.8%)
(h) Improve mental health	9 (29.0%)
(i) I do not know^∗^	7 (22.6%)
(4) Which of the following risk factors can lead to the occurrence and development of coronary heart disease? (check all that apply)	
(a) Hypertension	259 (79.7%)
(b) Hyperlipidemia	208 (64.0%)
(c) Hyperglycemia	202 (62.2%)
(d) Overweight/obesity	162 (49.8%)
(e) Tobacco use	160 (49.2%)
(f) Excessive drinking	145 (44.6%)
(g) Lack of exercise	118 (36.3%)
(h) Excessive psychological stress	110 (33.8%)
(i) I do not know^∗^	37 (11.4%)
(5) Do you agree that the occurrence and development of coronary heart disease can be controlled?	
(a) Yes	205 (63.1%)
(b) No^∗^	30 (9.2%)
(c) I do not know^∗^	88 (27.1%)
(6) How frequently do coronary heart disease patients need to assess their lipids?	
(a) Every 1-3 months^∗^	24 (7.4%)
(b) 3-6 months	102 (31.4%)
(c) 6-9 months^∗^	49 (15.1%)
(d) 9-12 months^∗^	14 (4.3%)
(e) Unknown^∗^	135 (41.5%)
(7) Which of the following practices help control blood lipids? (check all that apply)	
(a) Reduce the intake of saturated fatty acids (e.g., lard, cream)	251 (77.2%)
(b) Reduce high cholesterol intake (e.g., animal guts and egg yolks)	251 (77.2%)
(c) Eat more foods that can lower low-density lipoprotein cholesterol (e.g., fish)	170 (52.3%)
(d) Weight loss	150 (46.2%)
(e) Increase physical activity	136 (41.8%)
(f) Take lipid-lowering drugs	159 (48.9%)
(g) I do not know^∗^	26 (8.0%)
(8) Do patients with coronary heart disease need to measure blood pressure frequently?	
(a) Yes	282 (86.8%)
(b) No^∗^	13 (4.0%)
(c) I do not know^∗^	29 (8.9%)
(9) Which of the following statements about lowering blood pressure are true? (check all that apply)	
(a) Stop the medication after your blood pressure is controlled^∗^	259 (79.7%)
(b) Stick to moderate exercise	208 (64.0%)
(c) Increase intake of fresh vegetables and fruits	202 (62.2%)
(d) Antihypertensive drugs require lifelong use	162 (49.8%)
(e) Reduce mental stress	160 (49.2%)
(f) Stay in bed mainly^∗^	145 (44.6%)
(g) Gradually reduce your salt intake until you eat less than 6 grams a day	118 (36.3%)
(h) Limit drinking	110 (33.8%)
(i) I do not know^∗^	37 (11.4%)
(10) If you are diagnosed with diabetes, which of the following measures will help reduce blood sugar? (check all that apply)	
(a) Diet control	253 (77.8%)
(b) Proper exercise	211 (64.9%)
(c) Blood sugar monitoring	191 (58.8%)
(d) Receive diabetes-related health education	117 (36.0%)
(e) Use hypoglycemic drugs	164 (50.5%)
(f) I do not know^∗^	49 (15.1%)
(11) The dietary recommendations for patients with coronary heart disease are the following: (check all that apply)	
(a) Do not overeat	206 (63.4%)
(b) Increase potassium-rich foods (e.g., nuts, beans, bananas, and kelp)	111(34.2%)
(c) Reduce intake of fatty foods	275 (84.6%)
(d) Eat more fresh fruits and vegetables	258 (79.4%)
(e) Increase dietary fiber intake	205 (63.1%)
(f) Reduce salt intake	239 (73.5%)
(g) I do not know^∗^	19 (5.8%)
(12) The waist circumference of patients with coronary heart disease should be less than how many centimeters?	
(a) Male 90 cm/female 85 cm	45 (13.8%)
(b) Male 100 cm /female 95 cm^∗^	49 (15.1%)
(c) Male 110 cm/female 105 cm^∗^	17 (5.2%)
(d) Unknown^∗^	213 (65.5%)
(13) Do you know what the following measures should be taken against being overweight? (check all that apply)	
(a) Dietary control, lower calorie intake	289 (88.9%)
(b) Strengthening exercises	265 (81.5%)
(c) Use weight-loss medication^∗^	12 (3.7%)
(d) I do not know^∗^	12 (3.7%)
(14) Have you ever heard of secondary prevention of heart disease?	
(a) Yes	24 (7.4%)
(b) No^∗^	300 (92.3%)
(15) Have you heard about the need to use secondary preventive medications long-term in patients with coronary heart disease?	
(a) Yes	21 (6.5%)
(b) No^∗^	303 (93.2%)
(16) Do excessive stress and anxiety affect the recovery of coronary heart disease patients?	
(a) Yes	267 (82.2%)
(B) No^∗^	19 (5.8%)
(C) I do not know^∗^	38 (11.7%)
(17) Can exercise help reduce bad mood?	
(a) Yes	262 (80.6%)
(b) No^∗^	20 (6.2%)
(c) I do not know^∗^	42 (12.9%)
(18) Do patients with coronary heart disease need structured exercise after their condition is stabilized?	
(a) Yes	301 (92.6%)
(b) No^∗^	6 (1.8%)
(c) I do not know^∗^	16 (4.9%)
(19) Does proper exercise improve heart function?	
(a) Yes	296 (91.1%)
(b) No^∗^	6 (1.8%)
(c) I do not know^∗^	21 (6.5%)
(20) Which of the following statements is true for patients with coronary heart disease? (check all that apply)	
(a) The more the exercise, the better^∗^	3 (0.9%)
(b) Start with a small amount of exercise, increase gradually, and persist	303 (93.2%)
(c) It's good to have a heavy sweat^∗^	3 (0.9%)
(d) Even if you have discomfort during exercise, continue to exercise^∗^	5 (1.5%)
(e) I do not know^∗^	15 (4.6%)
(21) Which of the following types of exercise do you think patients with coronary heart disease can choose? (check all that apply)	
(a) Dumbbells	9 (2.8%)
(b) Jogging	139 (42.8%)
(c) Swimming	56 (17.2%)
(d) Walking	306 (94.2%)
(e) I do not know^∗^	Yes: 8 (2.5%)
(22) Which of the following methods can help you judge that the intensity of your activity has reached a suitable moderate range? (check all that apply)	
(a) Increase in heart rate by 20 to 30 beats/min after exercise compared to before exercise	102 (31.4%)
(b) Increase in heart rate by 40 to 50 beats/min after exercise compared to before exercise^∗^	22 (6.8%)
(c) Feel yourself breathing faster with exercise, but not short of breath	83 (25.5%)
(d) Dyspnea after exercise^∗^	6 (1.8%)
(e) The body sweats slightly after exercise	200 (61.5%)
(f) I do not know^∗^	65 (20.0%)
(23) How long do you think it is appropriate for patients with coronary heart disease to exercise at moderate intensity?	
(a) 10 minutes or so^∗^	58 (17.8%)
(b) 30-90 minutes	229 (70.5%)
(c) More than 120 minutes^∗^	2 (0.6%)
(d) I do not know^∗^	35 (10.8%)
(24) How many times per week is it recommended for patients with coronary heart disease to do the above moderate-intensity exercise?	
(a) <3 times^∗^	31 (9.5%)
(b) 3~7 times	237 (72.9%)
(c) >7 times^∗^	21 (6.5%)
(d) I do not know^∗^	35 (10.8%)
(25) Do you know what measures should be taken in case of chest discomfort or fatigue during exercise? (check all that apply)	
(a) Keep exercising^∗^	3 (0.9%)
(b) Immediately stop and rest on site	307 (94.5%)
(c) If the symptoms are not relieved after rest, take a nitroglycerin pill under the tongue. After 5 minutes, if it is still not relieved, take another pill. If the symptom still persists, call first aid	177 (54.5%)
(d) I do not know^∗^	4 (1.2%)
(26) If someone has a sleep problem, can it affect the development of coronary heart disease?	
(a) Yes	259 (79.7%)
(b) No^∗^	16 (4.9%)
(C) I do not know^∗^	49 (15.1%)
(27) When insomnia occurs, which of the following measures can be undertaken to improve sleep? (check all that apply)	
(a) Identify the causes of insomnia and take targeted measures	141 (43.4%)
(b) Follow your doctor's advice as soon as possible to use sedative sleeping pills	128 (39.4%)
(c) Professionals conduct psychological counseling	100 (30.8%)
(d) Appropriate exercise	81 (24.9%)
(e) I do not know^∗^	82 (25.2%)
(28) Is regular follow-up with your doctor necessary?	
(a) Yes	310 (95.4%)
(b) No^∗^	5 (1.5%)
(c) I do not know^∗^	9 (2.8%)
(29) How many beats per minute is ideal for your heart rate?	
(a) <55/min^∗^	2 (0.6%)
(b) 55~60/min	89 (27.4%)
(c) 60~70/min^∗^	137 (42.2%)
(d) >70/min^∗^	40 (12.3%)
(e) I do not know^∗^	56 (17.2%)
Total score (mean ± standard deviation)	45.45 ± 11.5

^˄^Patients who answered “no” they had not heard of CR to question 1 were directed to skip to question 4. Therefore, the percentage reported is based on the available denominator for these items. ^∗^Incorrect responses.

**Table 3:** Exploratory factor analysis of the Chinese/Mandarin version of CRBS, *n* = 380.

Factor					
Item	CR need	External logistical factors	Time conflicts	Program and health system-level factors	Comorbidities/functional status
18. … I can manage my heart problem on my own	0.760				
6…I do not need cardiac rehab (e.g., feel well, heart problem treated, not serious)	0.736				
21…I prefer to take care of my health alone, not in a group	0.734				
17… many people with heart problems do not go, and they are fine	0.703				
7…I already exercise at home, or in my community	0.579	0.368			
5…I did not know about cardiac rehab (e.g., doctor did not tell me about it)	0.392	0.369			
3…of transportation problems (e.g., access to car, public transportation)		0.809			
1…of distance (e.g., not located in your area, too far to travel)		0.765			
2…of cost (e.g., parking, gas)		0.743			
8…severe weather		0.559	0.376		
11…of time constraints (e.g., too busy, inconvenient class time)			0.821		
10…travel (e.g., holidays, business, cottage)			0.800		
12…of work responsibilities			0.719		
4…of family responsibilities (e.g., caregiving)		0.415	0.467		
19… I think I was referred, but the rehab program did not contact me				0.745	
20…it took too long to get referred and into the program	0.359			0.741	
16…my doctor did not feel it was necessary				0.613	
14…other health problems prevent me from going				0.307	0.783
15…I am too old					0.739
13…I do not have the energy					0.558
9…I find exercise tiring or painful	0.320	0.312	0.345		0.356
Variance explained	30.5%	8.7%	7.5%	7.1%	5.4%
Eigenvalues	6.41	1.84	1.59	1.50	1.14
Reliability	0.823	0.820	0.773	0.674	0.676

CRBS: Cardiac Rehabilitation Barriers Scale.

**Table 4:** Mean CRBS item and subscale scores (standard deviation) by CR participation.

Item	Total sample (*n* = 380)	Participated in CR (*n* = 19)	Did not participate (*n* = 361)	*p*
1…of distance (e.g., not located in your area, too far to travel)	3.6 ± 1.0	2.8 ± 1.2	3.7 ± 0.9	0.013
2…of cost (e.g., parking, gas)	3.1 ± 1.1	2.2 ± 1.0	3.2 ± 1.1	<0.001
3…of transportation problems (e.g., access to car, public transportation)	3.4 ± 1.1	2.6 ± 1.1	3.5 ± 1.0	0.001
4…of family responsibilities (e.g., caregiving)	3.1 ± 1.1	2.2 ± 1.0	3.2 ± 1.1	0.001
5…I did not know about cardiac rehab (e.g., doctor did not tell me about it)	3.5 ± 1.0	2.1 ± 1.1	3.6 ± 1.0	<0.001
6…I do not need cardiac rehab (e.g., feel well, heart problem treated, not serious)	2.8 ± 1.1	2.1 ± 0.9	2.8 ± 1.1	0.008
7…I already exercise at home, or in my community	3.0 ± 1.1	2.2 ± 1.0	3.0 ± 1.0	0.002
8…severe weather	3.5 ± 1.0	2.7 ± 1.3	3.5 ± 0.9	0.013
9…I find exercise tiring or painful	3.0 ± 1.0	2.2 ± 1.0	3.1 ± 1.0	0.001
10…travel (e.g., holidays, business, cottage)	2.9 ± 1.1	2.5 ± 1.2	3.0 ± 1.0	0.058
11…of time constraints (e.g., too busy, inconvenient class time)	3.0 ± 1.1	2.6 ± 1.1	3.1 ± 1.1	0.076
12…of work responsibilities	2.5 ± 1.0	2.2 ± 0.9	2.5 ± 1.0	0.187
13…I do not have the energy	3.0 ± 1.1	2.1 ± 0.9	3.0 ± 1.1	<0.001
14…other health problems prevent me from going	3.0 ± 1.0	3.2 ± 1.2	2.9 ± 1.0	0.358
15…I am too old	3.0 ± 1.1	2.4 ± 1.1	3.0 ± 1.1	0.014
16…my doctor did not feel it was necessary	3.1 ± 1.0	2.7 ± 1.2	3.2 ± 1.0	0.047
17… many people with heart problems do not go, and they are fine	3.0 ± 1.0	2.4 ± 1.2	3.0 ± 1.0	0.013
18… I can manage my heart problem on my own	2.8 ± 1.0	2.1 ± 0.9	2.8 ± 1.0	0.003
19… I think I was referred, but the rehab program did not contact me	2.9 ± 1.0	2.0 ± 0.8	3.0 ± 1.0	<0.001
20…it took too long to get referred and into the program	3.0 ± 0.9	2.0 ± 0.8	3.0 ± 0.9	<0.001
21…I prefer to take care of my health alone, not in a group	2.8 ± 1.0	2.2 ± 1.0	2.8 ± 1.0	0.015
Total mean CRBS score	3.04 ± 0.56	2.35 ± 0.71	3.08 ± 0.55	<0.001
Factor 1: CR need	2.98 ± 0.67	2.16 ± 0.88	3.01 ± 0.64	<0.001
Factor 2: external logistical factors	3.28 ± 0.69	2.38 ± 0.86	3.32 ± 0.67	<0.001
Factor 3: time conflicts	3.01 ± 0.71	2.39 ± 0.82	3.05 ± 0.71	<0.001
Factor 4: program and health system-level factors	2.98 ± 0.71	2.46 ± 0.59	3.03 ± 0.71	0.001
Factor 5: comorbidities	2.98 ± 0.75	2.46 ± 0.76	3.01 ± 0.75	0.003

CRBS: Cardiac Rehabilitation Barriers Scale; CR: cardiac rehabilitation. Note: *p* values are based on independent sample *t*-test results.

## Data Availability

Data is available from the corresponding author upon reasonable request.

## Supplementary Materials

Supplementary Materials Supplementary Table 1: Pearson's correlation coefficient showing association between with individual CRBS item scores with CRIAQ total scores. Supplemental Appendix: the CRBS scale https://sgrace.info.yorku.ca/cr-barriers-scale/crbs-instructions-and-languages-translations.
