# Supplementary material for: Translation, Cross-Cultural Adaptation, and Psychometric Validation of the Chinese/Mandarin Cardiac Rehabilitation Barriers Scale (CRBS-C/M)
Source: Rehabil Res Pract. 2021 Jun 17;2021:5511426. doi: 10.1155/2021/5511426 (PMC8233091; doi:10.1155/2021/5511426)
Supplement: Supplementary Materials — Supplementary Table 1: Pearson's correlation coefficient showing association between with individual CRBS item scores with CRIAQ total scores. Supplemental Appendix: the CRBS scale https://sgrace.info.yorku.ca/cr-barriers-scale/crbs-instructions-and-languages-translations. [file 5511426.f1.zip › 5511426.f1/CRBS_Chinese version from Lindsey_manuscript-AF_vRehRes&Pract-suppl w chinese scale.docx]

**心脏康复障碍量表**

以下问题将涉及一些影响您参与心脏康复活动的因素。无论您是否参加过心脏康复项目，请您回答以下所有问题。

- 您参加过心脏康复项目吗 是□ 否□
- 如果您参加过心脏康复计划，但是缺席过几次或者您从来没有参加过心脏康复计划，假设您有机会参加，您认为将阻碍您参与的原因为

1. 距离（如：康复场所不在我的居住社区，或者路程太远等）
2. 非常不同意 B.不同意 C.不确定 D.同意 E,非常同意
3. 花费（如：交通费、公交地铁费）
4. 非常不同意 B.不同意 C.不确定 D.同意 E,非常同意
5. 交通问题（如：是否有私家车，公共交通是否便捷等）
6. 非常不同意 B.不同意 C.不确定 D.同意 E,非常同意

4. 家庭责任（如：需要照顾家庭）

1. 非常不同意 B.不同意 C.不确定 D.同意 E,非常同意

5．我没听过心脏康复（如：医护人员未告知过我有心脏康复）

1. 非常不同意 B.不同意 C.不确定 D.同意 E,非常同意

6. 我不需要心脏康复（如：自我感觉身体良好，自我感觉心脏问题已缓解或者不是很严重等）

1. 非常不同意 B.不同意 C.不确定 D.同意 E,非常同意

7. 我已经在家或者在社区进行运动锻炼

1. 非常不同意 B.不同意 C.不确定 D.同意 E,非常同意
2. 天气恶劣
3. 非常不同意 B.不同意 C.不确定 D.同意 E,非常同意

9. 我觉得锻炼较累或者较痛苦

1. 非常不同意 B.不同意 C.不确定 D.同意 E,非常同意

10. 我要外出（如：假期、出差、郊游等）

1. 非常不同意 B.不同意 C.不确定 D.同意 E,非常同意

11. 时间受限（如：太忙、康复时间不合适）

1. 非常不同意 B.不同意 C.不确定 D.同意 E,非常同意

12. 我要上班

1. 非常不同意 B.不同意 C.不确定 D.同意 E,非常同意

13. 我没有精力

1. 非常不同意 B.不同意 C.不确定 D.同意 E,非常同意

14. 我有其他健康问题，不能参加心脏康复项目(如:_______________)

1. 非常不同意 B.不同意 C.不确定 D.同意 E,非常同意

15. 我年龄太大

1. 非常不同意 B.不同意 C.不确定 D.同意 E,非常同意

16．我的主管医生认为我不需要参加心脏康复项目

1. 非常不同意 B.不同意 C.不确定 D.同意 E,非常同意
2. 尽管很多心脏病患者没有参加心脏康复项目，他们也没什么问题

A.非常不同意 B.不同意 C.不确定 D.同意 E,非常同意

1. 我有能力处理好我的心脏问题
2. 非常不同意 B.不同意 C.不确定 D.同意 E,非常同意

19. 我愿意参加心脏康复项目，但是至今康复项目组人员没有联系过我

A.非常不同意 B.不同意 C.不确定 D.同意 E,非常同意

20. 等待纳入心脏康复项目耗时太长

A.非常不同意 B.不同意 C.不确定 D.同意 E,非常同意

21. 我更倾向于自我康复，不愿意参加集体康复活动

A.非常不同意 B.不同意 C.不确定 D.同意 E,非常同意
