# Supplementary material for: Translation, Cross-Cultural Adaptation, and Psychometric Validation of the Chinese/Mandarin Cardiac Rehabilitation Barriers Scale (CRBS-C/M)
Source: Rehabil Res Pract. 2021 Jun 17;2021:5511426. doi: 10.1155/2021/5511426 (PMC8233091; doi:10.1155/2021/5511426)
Supplement: Supplementary Materials — Supplementary Table 1: Pearson's correlation coefficient showing association between with individual CRBS item scores with CRIAQ total scores. Supplemental Appendix: the CRBS scale https://sgrace.info.yorku.ca/cr-barriers-scale/crbs-instructions-and-languages-translations. [file 5511426.f1.zip › 5511426.f1/CRBS_Supplemental Table 1 (1).docx]

**Supplemental Table 1: Pearson’s correlation coefficient showing association between individual CRBS item scores with CRIAQ total scores.**

| CRBS Item | Correlation coefficient | p |
| --- | --- | --- |
| 1…of distance (e.g., not located in your area, too far to travel) | -0.221 | <0.001 |
| 2…of cost (e.g., parking, gas) | -0.333 | <0.001 |
| 3…of transportation problems (e.g., access to car, public transportation) | -0.262 | <0.001 |
| 4…of family responsibilities (e.g., caregiving) | -0.227 | <0.001 |
| 5…I didn’t know about cardiac rehab (e.g., doctor didn’t tell me about it) | -0.375 | <0.001 |
| 6…I don’t need cardiac rehab (e.g., feel well, heart problem treated, not serious) | -0.259 | <0.001 |
| 7…I already exercise at home, or in my community | -0.241 | <0.001 |
| 8…severe weather | -0.180 | 0.001 |
| 9…I find exercise tiring or painful | -0.297 | <0.001 |
| 10…travel (e.g., holidays, business, cottage) | -0.116 | 0.033 |
| 11…of time constraints (e.g., too busy, inconvenient class time) | -0.106 | 0.051 |
| 12…of work responsibilities | -0.070 | 0.196 |
| 13…I don’t have the energy | -0.218 | <0.001 |
| 14…other health problems prevent me from going ( | -0.086 | 0.113 |
| 15…I am too old | -0.241 | <0.001 |
| 16…my doctor did not feel it was necessary | -0.187 | 0.001 |
| 17… many people with heart problems don’t go, and they are fine | -0.321 | <0.001 |
| 18… I can manage my heart problem on my own | -0.232 | <0.001 |
| 19… I think I was referred, but the rehab program didn’t contact me | -0.275 | <0.001 |
| 20…it took too long to get referred and into the program | -0.306 | <0.001 |
| 21…I prefer to take care of my health alone, not in a group | -0.186 | 0.001 |
| Total CRBS | -0.402 | <0.001 |

*CRBS= Cardiac Rehabilitation Barriers Scale, CRIAQ= Cardiac Rehabilitation Information Awareness Questionnaire*
